# Transcriptional and H3K27ac related genome profiles in oral squamous cell carcinoma cells treated with metformin

**DOI:** 10.7150/jca.63234

**Published:** 2022-03-21

**Authors:** Shan Liu, Congyu Shi, Xiaoru Hou, Xudong Tian, Chunjie Li, Xiangrui Ma, Xiaoyi Wang, Pan Gao

**Affiliations:** 1 State Key Laboratory of Oral Diseases & National Clinical Research Center for Oral Diseases & Department of Head and Neck Oncology, West China Hospital of Stomatology, Sichuan University, Chengdu, China.; 2 Department of Oral and Maxillofacial Surgery, Binzhou Medical University Hospital, Binzhou, China.; 3 State Key Laboratory of Oral Diseases & National Clinical Research Center for Oral Diseases & Department of General and Emergency Dentistry, West China Hospital of Stomatology, Sichuan University, Chengdu, China.

**Keywords:** Metformin, oral squamous cell carcinoma, H3K27ac, H3K27me3, reprogrammed cancer regulation

## Abstract

**Objectives:** Metformin, a first-line drug that has been used for type 2 diabetes treatment, recently attracts broad attention for its therapeutic effects on diverse human cancers. However, its effect and the underlying mechanisms on oral squamous cell carcinoma (OSCC) are not well known.

**Materials and Methods:** OSCC cells were used to evaluate the effect of metformin on cell proliferation and colony formation *in vitro*. Tumor formation assay in nude mice was conducted to assess the effect of metformin *in vivo*. Western blotting and immunohistochemistry stain were performed to investigate the effect of metformin on the expression of acetylation at lysine 27 of histone H3 (H3K27ac) and methylation at lysine 27 of histone H3 (H3K27me3) *in vitro* and* in vivo*. RNA-seq and ChIP-seq were performed to explore the genome profile to metformin treatment in OSCC cells.

**Results:** Metformin inhibited OSCC cell proliferation and colony formation *in vitro*, as well as OSCC growth *in vivo*. Metformin increased the global H3K27ac modification *in vitro*. Transcriptome analysis suggested that metformin mainly downregulated pluripotency stem cell pathway, development involved pathways and upregulated cytokine and inflammatory pathways. Additionally, H3K27ac was involved in transcription, DNA repair and replication in metformin-treated OSCC cells.

**Conclusions:** Metformin inhibits OSCC growth concomitant upregulated global level of H3K27ac *in vitro*. This study provides insights into the molecule and epigenome basis on application of metformin in OSCC treatment, and highlights the underlying mechanisms of reprogrammed cancer regulation and epigenetic histone modification.

## Introduction

Oral cancer, with rising incidence in all age groups, has been a growing global problem and a top cause of mortality in some regions 1, 2. Oral squamous cell carcinoma (OSCC) is the most common type of oral cancer 1, 2. A multidisciplinary approach has been advocated for oral cancer treatment with improvement of prognosis. Neoadjuvant chemotherapy and palliative chemotherapy are potential and effective strategies to improve prognosis 3. Although, the combination of traditional therapies and immunotherapy in oral cancer is promising, the current statics are always accompanied by obvious adverse effects 4-6. Thus, it will be beneficial to apply safe chemotherapies with rare side effects.

Metformin, being safe and off patent, has been proven to be a promising anti-cancer drug for many malignancies in clinical studies 7. It has been reported that metformin reduces oral cancer risk in diabetics 8. More interestingly, metformin can not only work synergistically with traditional therapies but also rescue the therapeutic resistance 9-13. The canonical anti-cancer mechanism of metformin is that it causes energy starvation with activated AMPK and inhibited mTOR* in vitro* and* in vivo* 7. In addition, histone modification attributes to its anti-cancer effect by regulating gene expression 14, 15. Metformin induces an indirect inhibition on histone deacetylases (HDAC) and decreases the level of H3K27me3 by disrupting the catalytic activity of polycomb repressive complex 2 (PRC2) 14, 16. The decreased H3K27me3 is concurrent with the upregulated H3K27ac, which results in activated transcription of the genome 17. It remains less exploration on the association between anti-cancer effect of metformin with H3K27ac or H3K27me3. Hence, our study aimed to reveal the potential role of metformin in OSCC treatment, and the underlying mechanisms related to H3K27me3 and H3K27ac modification.

Here, we showed the anti-cancer effect of metformin on OSCC with the globally increased H3K27ac level both* in vitro*. We studied the transcriptome and epigenetic H3K27ac signatures in metformin-treated OSCC cells. Our transcriptome data identified differentially expressed genes (DEGs) and were performed enrichment analysis, in which possible therapeutic and resistant profiles were identified. Furthermore, combined analysis of RNA-seq and ChiP-seq data showed the possible active or repressive effects of H3K27ac in different metformin-treated OSCC cell lines. Taken together, it is suggested that metformin has a potential effect to inhibit OSCC growth, the mechanism of which might be associated with epigenetic H3K27ac modification partly.

## Materials and methods

### Cell culture and chemicals

Cal27, HSC-2 and SCC25 were obtained from State Key Laboratory of Oral Diseases, Sichuan University, China. HaCaT was purchased from Procell(Wuhan, China). Cells were cultured in high glucose DMEM (Gibco, USA), supplemented with 10% fetal bovine serum (FBS, Gibco, USA) and 1% penicillin and streptomycin (100 μg/ml) (Hyclone, USA). Metformin was purchased from Sigma-Aldrich (1115-70-4, Germany). GSK126 was purchased from Selleck (S7061, USA).

### Cell proliferation assay

Cells (3 × 10^3^) were seeded into 96-well plates and treated with 0, 2.5, 5, 10, 20 and 40 mM metformin for 24, 48, 72 and 96 h. The cells were incubated with Cell counting kit-8 (CCK8; Donjido, Japan) at 37 °C for 1.5 h following the manufacturer's instruction. The absorbance at 450 nm was detected by a microplate reader (Thermo, USA).

### Colony formation

Cells (500 cells per well) were seeded into 6-well plates and incubated overnight allowing to cell adherence. Then cells were treated with 0, 5 and 10 mM metformin for 14 days until the visible colonies were generated. After the fixation by 4% paraformaldehyde, colonies were stained with crystal violet for 5 min. The colonies were photographed by digital camera and counted under the phase-contrast microscope.

### Tumorigenesis

Cal27 cells (5×10^6^) in logarithm stage were suspended in 200 μl phosphate-buffered saline (PBS) and injected into the right armpit of 6-week-old BALB/C nude mice. When the tumors were palpable one week later after the injection, mice were randomly divided into the group treated with metformin and the other without metformin. For the treated group, metformin (200 μg/ml) was added into the drink water in a light-protected bottle and the control group were supplied with normal drink water. The animal weight, random blood glucose and tumor volume were detected until the tumor volume was up to 1,000 mm^3^. The tumor volume was calculated according to the formula: volume = (major diameter^2^×minor diameter) / 2. The mice were sacrificed after 3-week treatment and tissues were fixed with 4% paraformaldehyde for hematoxylin-eosin (HE) stain and immunohistochemistry (IHC) analysis.

### Immunohistochemistry

The IHC analysis was conducted according to the instruction of antibodies' manufacturers. The primary antibodies were as following: H3K27me3 (#9733, Cell Signaling Technology, USA), H3K27ac (#8173, Cell Signaling Technology, USA) and Ki67 (#AF1738, Beyotime, China). IHC scores were calculated as previously reported 18.

### Western blotting

Cells and tissues were lysed in RIPA (P0013B, Beyotime, China) supplemented with 1 mM PMSF (ST506, Beyotime, China) and 0.01 mM phosphatase inhibitors (P1260, Solarbio, China). Samples with 20 ug protein in each group were separated by 10% SDS-PAGE and electrotransferred to PVDF membranes (Millipore, USA). The primary antibodies were used as following: β-actin (1:2000, 20536-1-AP, Protein tech, China), EZH2 (1:1000, #5246, Cell Signaling Technology, USA), SUZ12 (1:1000, #3737, Cell signaling Technology, USA), EED (1:1000, 16818-1-AP, Protein tech, China), Histone 3 (1:2000, 17168-1-AP Protein tech, China), H3K27me3 (1:1000, #9733, Cell Signaling Technology, USA), and H3K27ac (1:1000, #8173, Cell Signaling Technology, USA). After incubated with horseradish peroxidase-conjugated secondary antibody (1:2000, #7074, Cell Signaling Technology, USA), the blots were visualized with chemiluminescent substrate (US Everbright Inc., China) by Image Lab (Bio-Rad, USA).

### RNA-seq

OSCC cells, Cal27 and HSC-2, were treated with or without 10 mM metformin for 6 days. According to the methods in previous study 19, cells (1×10^6^) were lysed with Trizol reagent (Invitrogen). The RNA quality was checked by Bioanalyzer 2200 (Aligent). The RNA with RIN >8.0 is right for rRNA depletion. The cDNA libraries of each pooled RNA sample for single-end sequencing were prepared using lon Total RNA-Seq Kit v2.0 (Life Technologies) according to the manufacturer's instructions. The cDNA libraries were processed for the proton sequencing process and sequenced on Proton sequencers according to Ion PI Sequencing 200 Kit v2.0 (Life Technologies) by NovelBio Corp. Laboratory (China). FastQC with default parameter was applied to filter the adaptor sequence and removed the low-quality reads. The clean reads were aligned to the human reference genome sequence GRCH38 using the MapSplice program (v2.1.6). HTSeq was applied to calculate the counts of genes. RPKM/FPKM (Reads/Fragments per kilobase million reads) was used to standardize the expression data. Then DEseq2 algorithm was performed to filter the differentially expressed genes under the following criteria, ABS(Log2FC)>1 and FDR<0.05. Pathway analysis was used to find out the significant pathway of DEGs and annotated by KEGG (Kyoto Encyclopedia of Genes and Genomes) 20. Fisher's exact test was used to select the significant pathway. The resulting P values were adjusted using the BH FDR algorithm and threshold was set 0.05.

### ChIP-seq

OSCC cells, Cal27 and HSC-2, were cultured with or without 10 mM metformin for 6 days. Chromatin immunoprecipitation was performed according to previous report 21. Briefly, cells (1×10^8^) were cross-linked using 1% paraformaldehyde for 5 min and quenched with 125 mM glycine at room temperature. Chromatin fragments was generated between 100 and 750 bp. Then the solubilized chromatin fragments were immunoprecipitated with antibodies against H3K27me3 (#9733, Cell Signaling Technology, USA), H3K27ac (#8173, Cell Signaling Technology, USA) and Normal Rabbit IgG (#2729, Cell Signaling Technology, USA) as previously described. The ChIPed DNA fragments were used for sequence library using the Acegen DNA library Pre Kit (Acegen) according to manufacturer's protocol. The constructed libraries were analyzed by Aglient 2100 Bioanalyzer and finally sequenced on Illumina Novaseq 6000 using a 150×2 paired-end sequencing protocol. FastQC and Trimmomatic was used to perform quality control and trimming on the raw sequencing data. Clean reads were aligned to human genome (UCSC hg19) using Burrows Wheeler Aligner. Peak calling was performed with MACS2 software. Differentially enriched peaks were analyzed by Pepr software. The sequencing and data analysis were conducted by Novogene Bioinformatics Technology (China).

### RNA extraction and real-time PCR

Total RNA was extracted using trizol and transcribed to cDNA using Servicebio®RT First Strand cDNA Synthesis Kit (Servicebio, China). SYBR Green qPCR Master Mix (Servicebio, China) was performed for qPCR by real-time PCR system (CFX, Bio-Rad). Primers' information was listed in Table 1. The target genes relative mRNA expression was calculated by 2^-ΔΔCt^ method.

### Patient characteristics

The present study retrospectively reviewed the OSCC patients with type II diabetes from Jan 2015 to Dec 2018 at West China Hospital of Stomatology, Sichuan University. The inclusion criteria were as follows: primary OSCC undergoing surgery in our hospital, received no preoperative radiotherapy or chemotherapy; with complete surgical pathology data and follow-up information. Those were excluded with uncertain surgical pathology data, non-complete treatment, and incomplete information.

### Statistics analysis

All data are presented as mean ± SD. All experiments were conducted three times independently except for extra interpretation. One-way analysis of variance (ANOVA) was used to compare the difference among multiple groups and Student's t test was used to analyze the difference of two groups. K-M analysis followed by long-rank test was used to analyze the correlation between metformin and the prognosis of oral cancer patients.* P*<0.05 was set to be statistical significance.

## Results

### Metformin inhibited cell viability and cell colony formation in OSCC cells and normal cells

**Figure 1:**
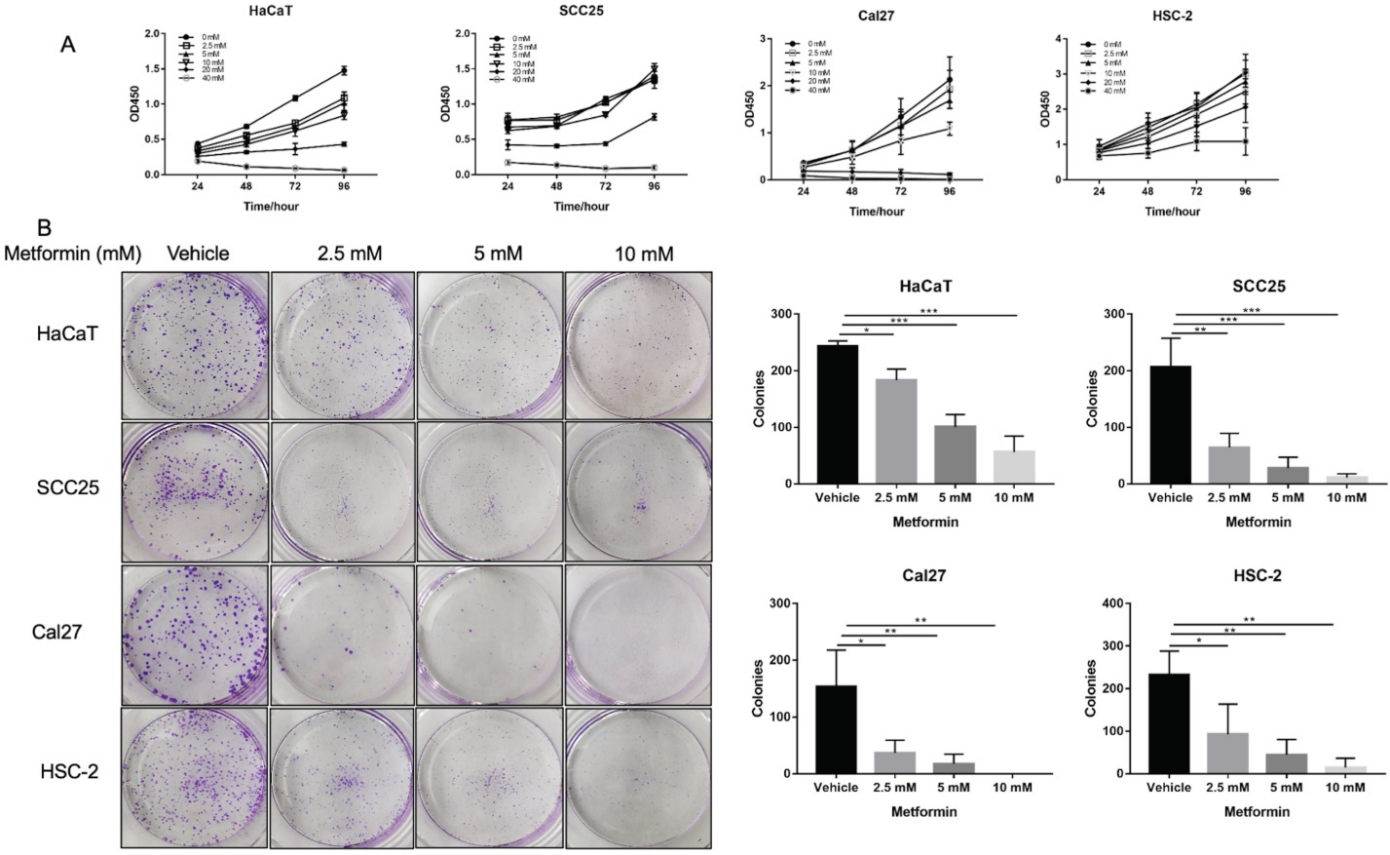
** Metformin inhibited OSCC growth *in vitro*. (A)** Cell proliferation of HaCaT, SCC25, Cal27 and HSC-2 cells induced by metformin was evaluated by CCK8 assay. Cells were treated by concentration gradient of metformin (vehicle, 2.5, 5, 10, 20, and 40 mM) for 24, 48, 72, and 96 h. **(B)** Proliferative ability of cells treated by metformin (vehicle, 2.5, 5 and 10 mM) was assessed by colony formation assay. Typical images (left) and quantification (right) in terms of colony numbers. Data are presented as mean ± SD from three independent experiments at least. *:* p*<0.05; **: *p*<0.01; ***: *p*<0.001.

Metformin inhibited cell viability of OSCC cells and normal control cells in a dose and time dependent manner. The cell viability of Cal27, HSC-2 and SCC25 was significantly inhibited by 10-, 20- and 40-mM metformin (*p*<0.05, Fig. 1A), while the cell viability of HaCaT was significantly inhibited even by 2.5- and 5-mM metformin (p<0.05, Fig. 1A). In line with the result of CCK8 assay, the colony formation of OSCC cells treated with metformin (5 and 10 mM) was significantly inhibited (*p*<0.05, Fig. 1B).

### Metformin inhibited tumorigenesis *in vivo*

**Figure 2:**
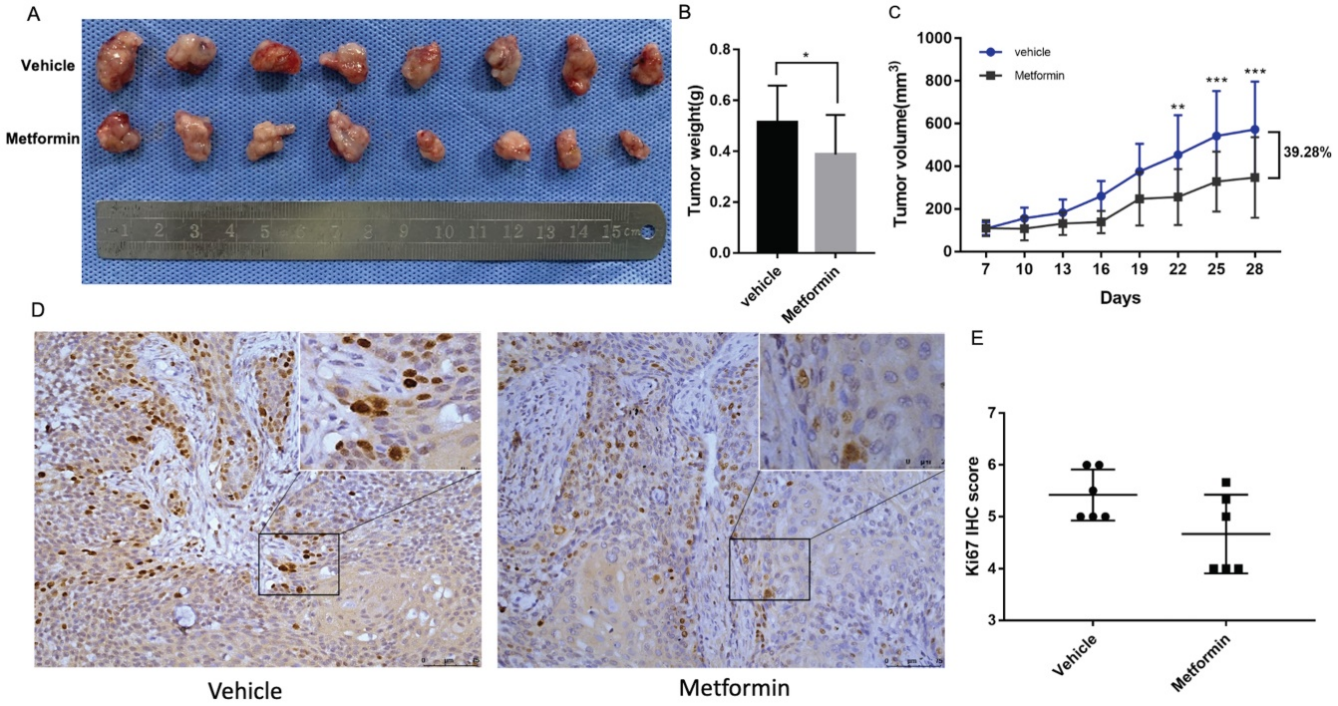
** Metformin inhibited OSCC tumor growth in nude mice. (A)** Macroscopic appearance of the dissected tumors from mice at the end of the experiment.** (B)** Tumor weight histogram. **(C)** Tumor growth curves. **(D)** Ki67 staining and **(E)** Ki67 IHC score.

Tumor formation assay in nude mice was performed to illustrate the anti-cancer effect of metformin *in vivo*. The tumor volumes and mice weights were recorded every three days after the tumors were palpable. After three-week treatment, the mice were euthanized and tumors were harvested and measured (Fig. 2A). At the end of the experiment, the tumor weight and volume of mice treated with metformin was 20% and 39.28%, respectively less than that of control group (*p*<0.05, Fig. 2B and 2C). Consistent with the decreased tumor weight and volume, ki67 score tended to be lower in tumor tissues of mice treated with metformin than that in control group (p>0.05, Fig. 2D and 2E).

### Metformin upregulated global level of H3K27ac in OSCC cell lines

**Figure 3:**
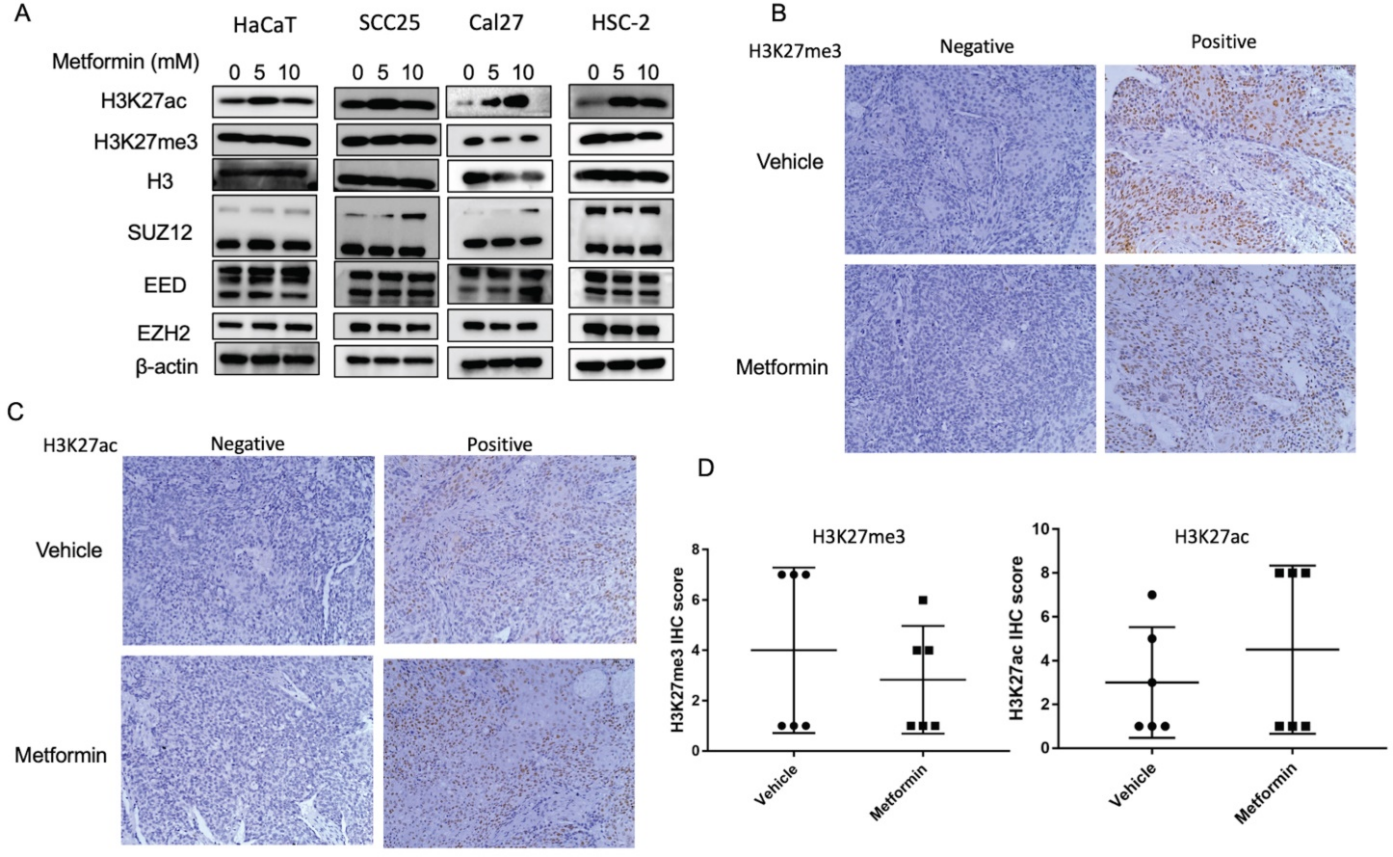
** Metformin upregulated global level of H3K27ac *in vitro*. (A)** Cells treated with metformin (vehicle, 5 and 10 mM) for 6 days were subjected to western blotting analysis. H3 served as the reference protein for H3K27ac and H3K27me3, and β-actin was used as reference protein for EZH2, EED and SUZ12. **(B)** Tumor tissues of nude mice were sectioned to conduct immunohistochemistry stain for antibody of H3K27me3. **(C)** Tumor tissues of nude mice were sectioned to conduct immunohistochemistry stain for antibody of H3K27ac. **(D)** IHC scores of H3K27me3 (left) and H3K27ac (right).

To explore the effect of metformin on H3K27ac and H3K27me3, cells and tumor tissues harvested from mice treated with or without metformin were used to detect the expression of H3K27ac and H3K27me3. As shown in Fig. 3A, HaCaT, SCC25, Cal27 and HSC-2 treated with metformin showed higher expression of H3K27ac than that of control group. Meanwhile, our data showed that metformin had no effect on the expression of EZH2, SUZ12 and EED (Fig. 3A). However, metformin had no stable effect on the expression of H3K27me3 and H3K27ac *in vivo* (Fig. 3B, 3C and 3D).

### Transcriptome profile to metformin treatment in OSCC cells

**Figure 4:**
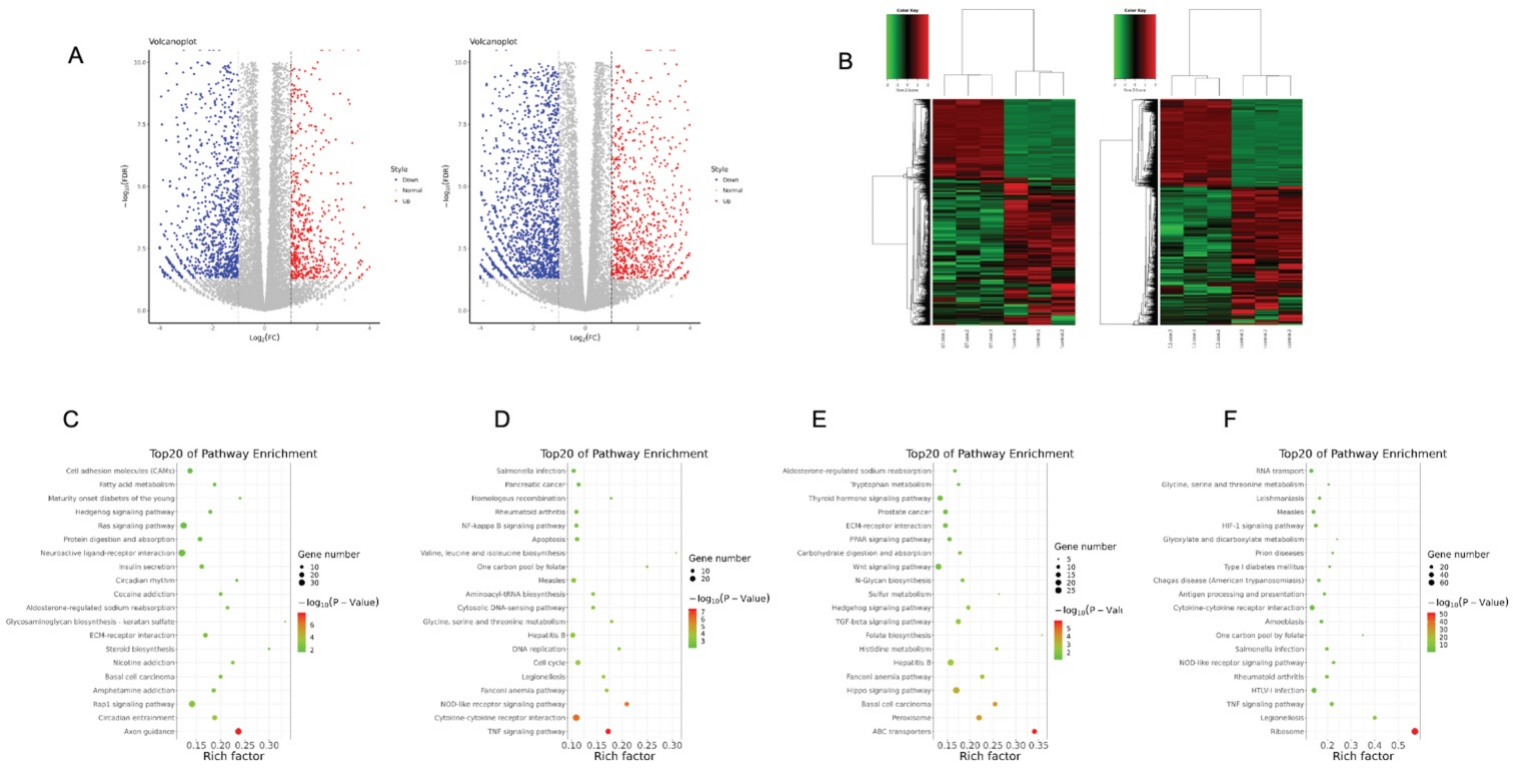
** Transcriptome profile of OSCC cell lines treated with metformin.** Volcano map (**A**) and gene-wise hierarchical clustering heatmap (**B**) of genome in response to metformin treatment in Cal27 and HSC-2 cell lines. **(C and D)** Enrichment pathway of downregulated (C) and upregulated (D) DEGs in Cal27. **(E and F)** Enrichment pathway of downregulated (E) and upregulated (F) DEGs in HSC-2.

**Figure 5:**
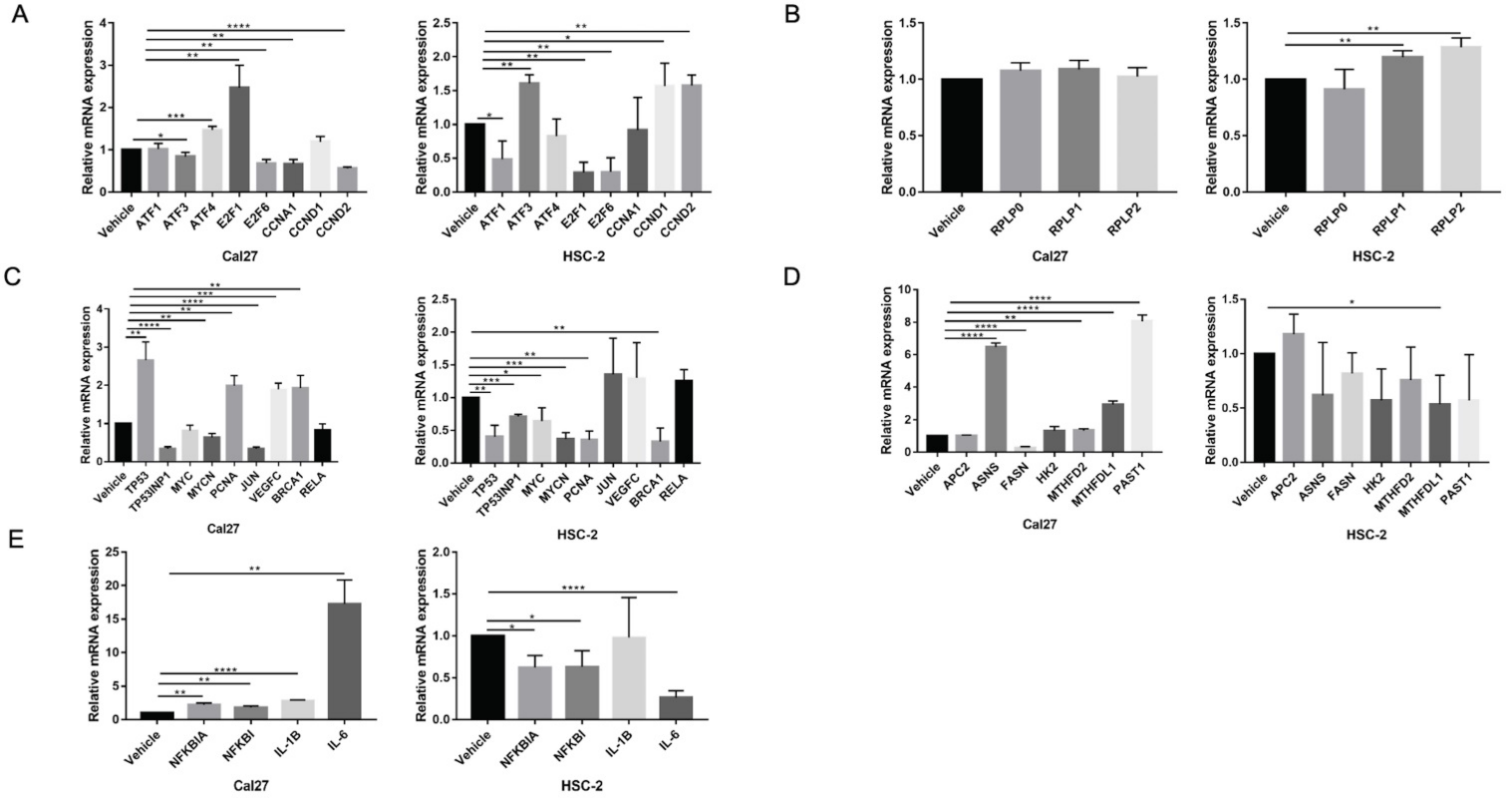
** The verification of dual effect of metformin on transcripts in OSCC cells treatment. (A)** Cell cycle regulation genes. **(B)** Ribosome genes. **(C)** The oncogenes and tumor suppressors. **(D)** Cell metabolism genes. **(E)** Cytokines expression.

To elucidate gene expression level affected by metformin in OSCC cells, we performed RNA-seq in Cal27 and HSC-2 cell lines treated with and without 10 mM metformin for 6 days. There were three biological triplicates for each cell line. DEGs were identified based on metformin treatment (Fig. 4A and 4B). We respectively identified 2203 and 3241 DEGs in Cal27 and HSC-2. We analyzed downregulated and upregulated DEGs in Cal27 and HSC-2 separately. KEGG pathway analysis in Cal27 found that downregulated DEGs induced by metformin were mainly enriched on Axon guidance, ECM-receptor interaction, cell adhesion molecules, pluripotency stem cells, and lipid metabolism pathways (Fig. 4C); and upregulated DEGs were mainly on TNF signaling pathway, cytokine-cytokine receptor interaction, NOD-like receptor signaling pathway, apoptosis, cell cycle, and amino acid metabolism pathways (Fig. 4D). For HSC-2, downregulated DEGs were mainly enriched on ABC transporters, peroxisome, Hippo signaling pathway, Wnt signaling pathway, ECM-receptor interaction, and hormone regulation (Fig. 4E); and upregulated DEGs were mainly on Ribosome, cytokine-cytokine receptor interaction, HIF-1 signaling pathway, and amino acid metabolism (Fig. 4F). Our results verified that metformin could modulate transcripts involved in cell cycle, ribosome, tumor oncogenes and suppressors, cell metabolism and cytokines (Fig. 5A-5E).

### The genome profile of H3K27 modification in metformin-treated OSCC cell lines

**Figure 6:**
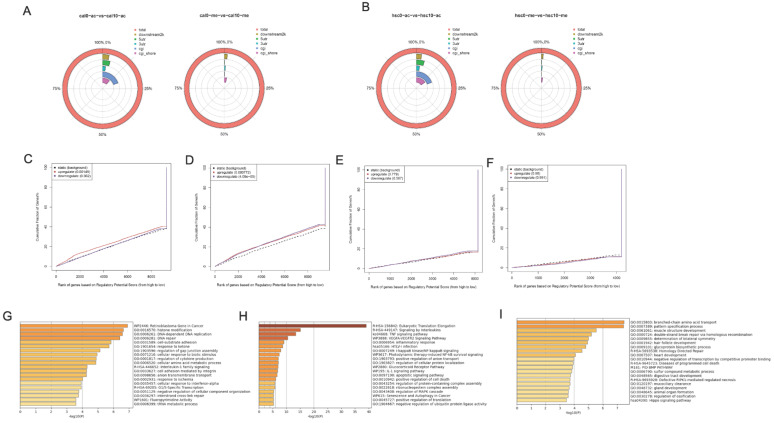
** Genome profile modified by H3K27ac in OSCC cell lines treated with metformin. (A and B)** The location of differential peaks in Cal27 (A) and HSC-2 (B). **(C and D)** BETA analysis predicted the function of H3K27ac on gene expression in Cal27 (C) and HSC-2 (D) treated with metformin. **(E and F)** BETA analysis predicted the function of H3K27me3 on gene expression in Cal27 (E) and HSC-2 (F) treated with metformin. **(G)** The pathway enrichment analysis based on upregulated target genes analyzed by BETA in Cal27. **(H and I)** The pathway enrichment analysis based on upregulated (H) and downregulated (I) target genes analyzed by BETA in HSC-2.

Although our results showed that metformin modified global H3K27ac level in OSCC cell lines, its underlying effect remains unclear. ChiP-seq was conducted to evaluate potential chromosome site modified by H3K27ac and H3K27me3 in Cal27 (Fig. 6A) and HSC-2 (Fig. 6B). Differential peaks were identified based on metformin treatment. Positive peaks were increased marks with metformin treatment, while negative peaks were decreased marks. ChIP-seq analysis on H3K27ac modification identified 2193 positive peaks and 2340 negative peaks in Cal27, while 5001 positive peaks and 111 negative peaks were identified in HSC-2. ChIP-seq analysis on H3K27me3 modification identified 783 negative peaks and 2381 positive peaks in Cal27, while 132 negative peaks and 3073 positive peaks in HSC-2. These results showed global histone modification status might not signify chromosome modification status. Therefore, we analyzed all differential peaks. Binding and Expression Target Analysis (BETA) is a software package that integrates ChIP-seq of transcript factors or chromatin regulators with differential gene expression data to infer direct target genes and collaborative factors 22. We used BETA online website, Cistrome, with default values to reveal if gene expression was affected by H3K27ac or H3K27me3 modification. A weak correlation was identified between RNA-seq and H3K27ac ChIP-seq data both in Cal27 and HSC-2 (Fig. 6C-6F). These results showed that H3K27ac had an activating effect on gene expression in Cal27 (Fig. 6C), while it had both repressive and activating effects on gene expression in HSC-2 (Fig. 6D). H3K27me3 had no effect on gene expression both in Cal27 (Fig. 6E) and HSC-2 (Fig. 6F). There were numerous possible direct targets of H3K27ac. Then enrichment analysis and gene ontology (GO) annotation on the direct predicted targets by BETA were analyzed by Metascape 23. The most enriched pathways in up regulated DEGs with H3K27ac modification of Cal27 were retinoblastoma gene in cancer, interleukin-1 family signaling and G1/S specific transcription, while the most enriched gene ontology (GO) annotations were histone modification, DNA-dependent DNA replication, and DNA-repair (Fig. 6G). The most enriched pathways in up regulated DEGs with H3K27ac modification of HSC-2 were Eukaryotic translation elongation, signaling by interleukins and TNF signaling pathway, while the most enriched GO annotations were inflammatory response, I-KappaB kinase/Nf-KppaB signaling, and positive regulation anion transport (Fig. 6H). The most enriched pathways in down regulated DEGs with H3K27ac modification of HSC-2 were homology directed repair, diseases of programmed cell death and BMP pathway, while the most enriched GO annotations were branched-chain amino acid transport, pattern specification process and muscle structure development (Fig. 6I).

**Figure 7:**
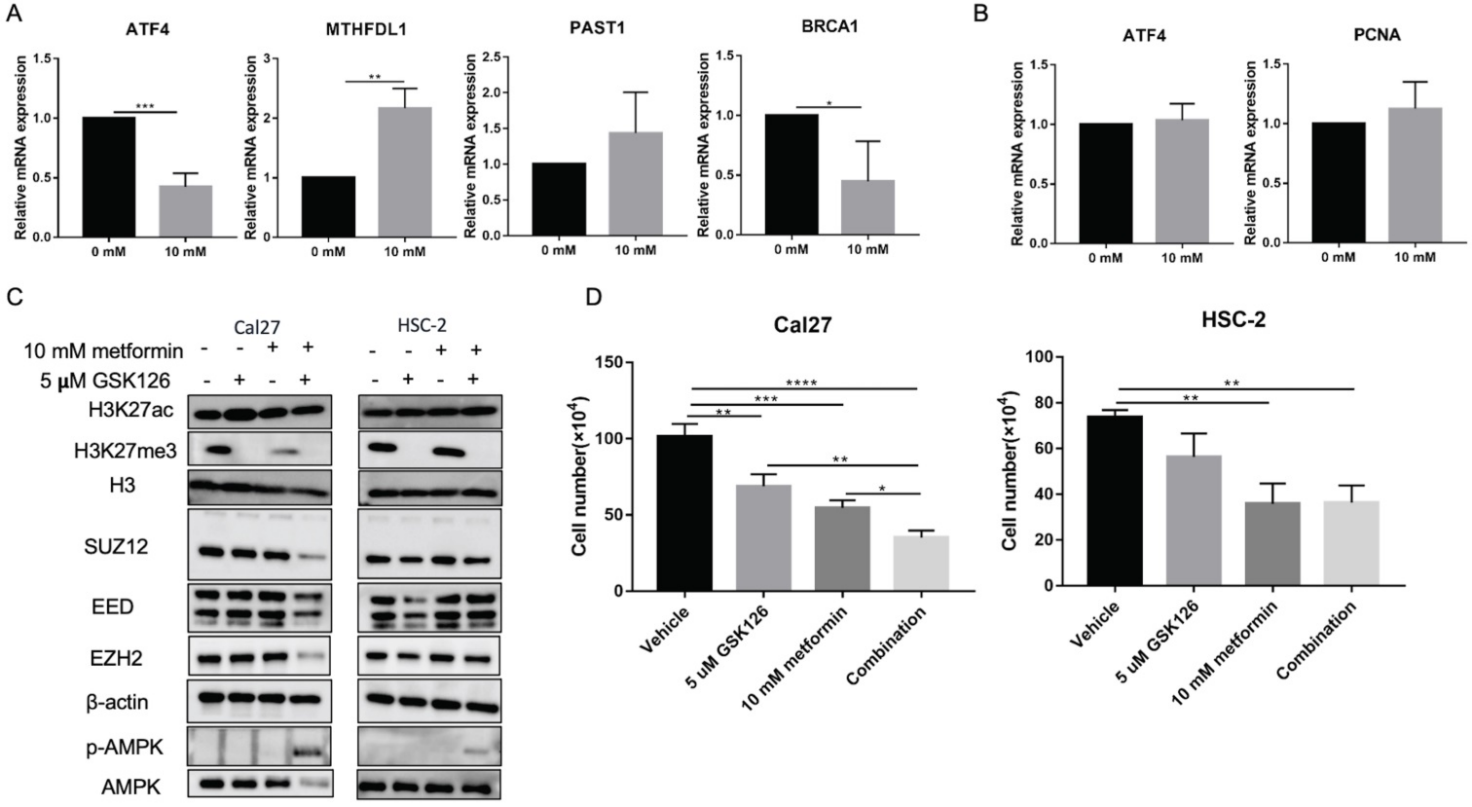
** GSK126 showed different regulation on transcripts from metformin. (A and B)** The effect of GSK126 on possible transcripts regulated by H3K27ac with metformin in Cal27 (A) and HSC-2 (B). **(C)** The western blot analysis of Cal27 and HSC-2 treated with metformin or (and) GSK126. **(D)** The cell growth of Cal27 and HSC-2 treated with metformin or (and) GSK126. Data are presented as mean ± SD from three independent experiments at least. *: *p*<0.05; **: *p*<0.01; ***: *p*<0.001.

Metformin has been demonstrated as one inhibitor for PRC2 catalytic activity 16, which also could be inhibited by GSK126. We used 5 uM GSK126 to treat cells and detect the possible transcripts modified by H3K27ac and H3K27me3 caused by metformin. The results showed that GSK126 could inhibit ATF4, BRCA1 and promote MTHDFL1 expression in Cal27, while it had no significant effect on the expression of PSAT1 in Cal27 (Fig. 7A). It had no inhibited effect on the transcript of ATF4 and PCNA in HSC-2 (Fig. 7B). GSK126 could upregulate the level of H3K27ac in Cal27 and HSC-2 while the combination of GSK126 and metformin had no synergistic effect on H3K27ac (Fig. 7C). Additionally, GSK126 had inhibitory effect on the cell growth of Cal27 and could enhance the inhibition of metformin on Cal27 growth (Fig. 7D).

### Metformin had no beneficial effect on disease-free survival and overall survival in OSCC patients

**Figure 8:**
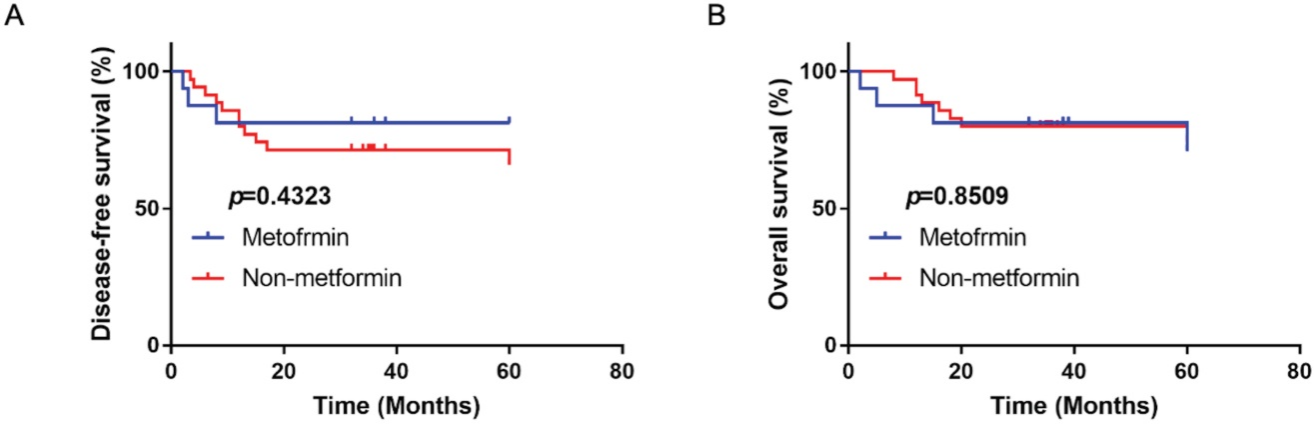
The disease-free survival (**A**) and overall survival (**B**) of OSCC patients with type II diabetes treated with or without metformin in clinic.

A total of 56 OSCC patients with type II diabetes were analyzed with the follow-up period from 3 years to 5 years. There were 16 patients using metformin as therapy for diabetes. Compared with patients with other anti-diabetic drugs, metformin had no significantly beneficial effect on disease-free and overall survival (*p*>0.05; Fig. 8).

## Discussion

### Metformin treatment caused reprogrammed cancer regulation

In this study, metformin was proved to have an anti-cancer effect on OSCC *in vivo* and *in vitro*, which was in line with the previous studies 24-29. The transcriptome prolife related to metformin treatment was identified by RNA-seq in OSCC cells. Different OSCC cell lines showed not exactly same response to metformin treatment, which indicated that there was heterogeneous response to metformin in different cell lines. Metformin downregulated pluripotency stem cell pathway and development related pathways, Wnt signaling pathway and Hedgehog pathway, which was associated with previous reported therapeutic targets of metformin 29. It indicated metformin might inhibit cancer progress. Additionally, metformin upregulated apoptosis and TNF signaling pathway to play its anti-cancer effect, which was in line with previous study 29. ABC transporter, associated with chemotherapy resistance and CSC phenotype, was downregulated in HSC-2, which might be one possible therapeutic effect of metformin 30. However, KEGG results showed cells' response to metformin was not only related to its anti-cancer effect. Ribosome was a place for protein biosynthesis and one enriched pathway of upregulated DEGs in HSC-2, indicating possible damage repair mechanism 31. Cytokine and immune response were upregulated in our study. Previous studies showed that metformin could reduce the expression of inflammatory cytokines in senescence and peripheral blood cells 32, 33. However, our study suggested that metformin increased the expression of pro-inflammatory and inflammatory cytokines, which might be linked to chemoresistance 34. In the study of acquired resistance to metformin of breast cancer cells, the most enriched category was chemokine signaling in upregulated genes 35. Moreover, metformin upregulated glycine, serine, and threonine metabolism, and one carbon pool by folate in these two cell lines, which was likely associated with NADH/NAD+ imbalance caused by metformin 36. Thus, our results indicated 6-day metformin treatment could induce therapeutic effects concomitant with possible resistant profile in culture system.

Collectively, these results suggested that 6-day metformin treatment reprogrammed cancer regulation in OSCC. Our study provides a global picture of transcriptome changes by metformin in OSCC cells, which indicates that metformin treatment causes possible anti-cancer effect and chemo-resistant profile at the same time.

### The controversial effect of metformin on global level of H3K27ac and H3K27me3

Current studies indicates that the effect of metformin on histone modification remains controversial. Galeidri et al. found metformin enhanced histone acetylation modification by activating AMPK in prostate and ovarian cancer cells 37. Fang et al. demonstrated that metformin enhanced histone acetylation level by regulating AMPK-CREB to improve depression 38. However, metformin corrected cancer-related histone acetylation by inhibiting mitochondrial biosynthetic capacity in cancer-prone human epithelial cells 39. Additionally, metformin simultaneously decreased the level of H3K27me3 in our study, which has been identified in breast cancer and ovarian cancer 16, 40. The decreased H3K27me3 level might attribute to inhibition or phosphorylation of PCR2 complex by activating AMPK 16, 40. Contrary to these findings, metformin restored global H3K27me3 level in aging syndromes with upregulated level of H3K27me3 41. Thus, it is difficult to draw a unified conclusion about the function of metformin on global level of H3K27ac and H3K27me3. Based on these findings, there is one possibility that metformin might affect the equilibrium of histone modification and the effect of metformin on histone modification might be dependent on cellular basic histone modification profile. Therefore, the histone modification might not fully elucidate metformin's anti-cancer mechanism, while histone modifications have been demonstrated to be predictive factors for cancer prognosis 42-46. Moreover, the discrepancy of H3K27ac and H3K27me3 expression *in vivo* and *in vitro* indicated metformin dose and treatment time might affect its effect on global level of H3K27ac and H3K27me3.

### H3K27ac modification induced by metformin associated with reprogrammed cancer regulation in OSCC cells

Histone modification has been demonstrated to be one regulatory factor of transcription, DNA repair, replication, stemness and changes in cell state, while the underlying mechanism remains elusive 47. Consistent with current knowledge, the combined analysis of ChIP-seq and RNA-seq suggested differential peaks modified by H3K27ac due to metformin were involved in DNA repair and transcription, collaborating transcriptional active of TFs. These results indicated that metformin treatment might affect transcription and chromosome stability by H3K27ac and its collaborators. Besides, our results suggested H3K27ac modification due to metformin treatment was involved in retinoblastoma gene in cancer, G1/S specific transcription, interleukins and inflammatory response, TNF signaling pathway, programmed cell death and BMP signaling pathway. As mentioned above, these pathways might contribute to therapeutic effects or induced resistance with metformin treatment. These results indicated histone modification might play dual effects in OSCC with metformin treatment, not solely beneficial or detrimental effect. The difference of GSK126 on transcripts from the effect of metformin indicated that H3K27ac might cooperate with other regulator modulating transcription. Furthermore, the level of H3K27ac was not coordinated with the inhibitory effect of treatment on cell growth (Fig. 7C and 7D).

There are some limitations in our study. We use millimolar metformin, which is over physiological concentration. There are some reasons. Firstly, our study is conducted in culture system with over physiological nutrients in tumors and current studies have demonstrated nutrient supply affected metformin efficacy 24-26. Secondly, we choose this concentration based on proliferation data to explore significant metformin response in OSCC cells. Nonetheless, our study provides a genome signature response to metformin in OSCC cells and highlights possible dual role of metformin treatment. Additionally, metformin effect is slight in our animal experiment and the discrepancy attributes to metformin treatment time, cell type, cell initial number, and metformin dose with other studies performing metformin in drink water 27, 29.

## Conclusion

Metformin changed the transcripts of OSCC involved in cell cycle, ribosome, tumor oncogenes and suppressors, cell metabolism, and cytokines, which may result from the regulation of chromosome stability modulated by H3K27ac and its collaborators. This study also provides insights into the molecule and epigenome basis to metformin treatment in OSCC and highlights the underlying beneficial or detrimental effects by reprogrammed cancer regulation and genome histone modification.

## Figures and Tables

**Table 1:** Primers were used in this study

Primers	Sequence	Primers	Sequence
Beta Actin F	CCAACCGCGAGAAGATGA	PCNA F	CCTGCTGGGATATTAGCTCCA
Beta Actin R	CCAGAGGCGTACAGGGATAG	PCNA R	CAGCGGTAGGTGTCGAAGC
ATF1 F	ACTCCCATCTATCAGACTAGCAG	JUN F	GCCAGGTCGGCAGTATAGTC
ATF1 R	CCTGGACTTGCCAACTGTAAG	JUN R	TCTGGACACTCCCGAAACAC
ATF3 F	CGCTGGAATCAGTCACTGTCAG	VEGFC F	GCTTCTTCTCTGTGGCGTGT
ATF3 R	CTTGTTTCGGCACTTTGCAGCTG	VEGFC R	TTTGCTTGCATAAGCCGTGG
ATF4 F	TTCTCCAGCGACAAGGCTAAGG	BRCA1 F	CTGAAGACTGCTCAGGGCTATC
ATF4 R	CTCCAACATCCAATCTGTCCCG	BRCA1 R	AGGGTAGCTGTTAGAAGGCTGG
E2F1 F	CATCCCAGGAGGTCACTTCTG	RELA F	TGAACCGAAACTCTGGCAGCTG
E2F1 R	GACAACAGCGGTTCTTGCTC	RELA R	CATCAGCTTGCGAAAAGGAGCC
E2F6 F	GCGAGGAAGTTACCCAGTCTC	APC2 F	GCCGACATCAACAGCAAGAAGG
E2F6 R	AGGATTCATGGCGAAGGCAG	APC2 R	CGCCTTGTTCTCTGTGCTGTGT
CCNA1 F	GCACACTCAAGTCAGACCTGCA	ASNS F	CATTACAACAGTTCGTGCTTCAG
CCNA1 R	ATCACATCTGTGCCAAGACTGGA	ASNS R	CACCACGCTATCTGTGTTCTT
CCND1 F	GCTGCGAAGTGGAAACCATC	FASN F	AAGGACCTGTCTAGGTTTGATGC
CCND1 R	CCTCCTTCTGCACACATTTGAA	FASN R	TGGCTTCATAGGTGACTTCCA
CCND2 F	GAGAAGCTGTCTCTGATCCGCA	HK2 F	GAGCCACCACTCACCCTACT
CCND2 R	CTTCCAGTTGCGATCATCGACG	HK2 R	CCAGGCATTCGGCAATGTG
RPLP0 F	TGGTCATCCAGCAGGTGTTCGA	MTHFD2 F	GATCCTGGTTGGCGAGAATCC
RPLP0 R	ACAGACACTGGCAACATTGCGG	MTHFD2 R	TCTGGAAGAGGCAACTGAACA
RPLP1 F	CAATGCCCTCATTAAAGCAGCCG	MTHFD1L F	CCCTTTGGTCGGAACGATGA
RPLP1 R	CCCTACATTGCAGATGAGGCTC	MTHFD1L R	GGTCCTGTGAGAGCCTTGTC
RPLP2 F	TCTTGGACAGCGTGGGTATCGA	PSAT1 F	ACAGGAGCTTGGTCAGCTAAG
RPLP2 R	CAGCAGGTACACTGGCAAGCTT	PSAT1 R	CATGCACCGTCTCATTTGCG
Tp53 F	GAGGTTGGCTCTGACTGTACC	NFKBIA F	CTCCGAGACTTTCGAGGAAATAC
Tp53 R	TCCGTCCCAGTAGATTACCAC	NFKBIA R	GCCATTGTAGTTGGTAGCCTTCA
TP53INP1 F	TTCCTCCAACCAAGAACCAGA	NFKBI F	GGTGCGGCTCATGTTTACAG
TP53INP1 R	GCTCAGTAGGTGACTCTTCACT	NFKBI R	GATGGCGTCTGATACCACGG
MYC F	GTCAAGAGGCGAACACACAAC	IL-1b F	TTCGAGGCACAAGGCACAA
MYC R	TTGGACGGACAGGATGTATGC	IL-1b R	CCATCATTTCACTGGCGAGC
MYCN F	TGATCCTCAAACGATGCCTTC	IL-6 F	ACTCACCTCTTCAGAACGAATTG
MYCN R	GGACGCCTCGCTCTTTATCT	IL-6 R	CCATCTTTGGAAGGTTCAGGTTG

## Abbreviations

OSCC: oral squamous cell carcinoma
H3K27ac: acetylation at lysine 27 of histone H3
HDAC: histone deacetylases
H3K27me3: tri-methylation at lysine 27 of histone H3
DEGs: differentially expressed genes
PRC2: polycomb repressive complex 2
FBS: fetal bovine serum
CCK8: Cell counting kit-8
PBS: phosphate-buffered saline
HE: hematoxylin-eosin
IHC: immunohistochemistry
RPKM/FPKM: Reads/Fragments per kilobase million reads
KEGG: Kyoto Encyclopedia of Genes and Genomes
ANOVA: One-way analysis of variance
BETA: Binding and Expression Target Analysis
GO: Gene ontology
